# It Takes Two to Tango: How the COVID-19 Vaccination Campaign in Israel Was Framed by the Health Ministry vs. the Television News

**DOI:** 10.3389/fpubh.2022.887579

**Published:** 2022-04-12

**Authors:** Anat Gesser-Edelsburg, Rana Hijazi, Ricky Cohen

**Affiliations:** ^1^School of Public Health, University of Haifa, Haifa, Israel; ^2^The Health and Risk Communication Lab, University of Haifa, Haifa, Israel; ^3^The Cheryl Spencer Department of Nursing, University of Haifa, Haifa, Israel

**Keywords:** COVID-19, vaccination campaign, media, news coverage, frame analysis, Israel

## Abstract

**Background:**

The internet has become a major resource in information transfer during COVID-19, and traditional means of communication are digitized and accessible online to the public at large.

**Objectives:**

This study seeks to examine how Israel's two main television news channels (Channel 12 and Channel 13) covered the Covid-19 vaccination campaign, compared to how the Ministry of Health ran the campaign.

**Methods:**

A qualitative study based on triangulation of online content analyses from three different sources: advertising campaigns, social media posts and reports on television news channels. The research sample included 252 reports from the newsrooms of Channel 13 (*n* = 151) and Channel N12 (*n* = 101), Israel's two leading news channels, all broadcast between December 1, 2020 and November 30, 2021. The sample also included posts from Israel Ministry of Health Facebook page and advertising campaigns from the Facebook page of the Israel Government Advertising Agency (LAPAM), which constructs advertising campaigns for the MOH (113 items).

**Results:**

The research findings reveal congruence between the way the MOH framed its vaccination campaign and news coverage of the vaccination issue. The vaccination campaign used three primary framing strategies: (1) positive framing (emphasizing the vaccine's advantages and stressing that the vaccine is safe and effective based on cost-benefit calculations and public health perspectives); (2) fear appeal strategy (conveying persuasive messages that seek to arouse fear through threats of impending danger or harm); (3) attribution of responsibility strategy (blaming the unvaccinated and targeting all those who criticized Israel's generic vaccination policy).

**Conclusion:**

As the watchdog of democracy, the news should function as a professional and objective source that criticizes government systems if necessary and strives to uncover the truth throughout the crisis. Public trust, which is so essential during such a crisis, can be achieved only if the news channels provide reports and meaningful journalistic investigations that challenge the system. By doing so, they can help fight conflicts of interest that divert management of the crisis from the professional health field to the political-economic arena.

## Introduction

The global media map at the outbreak of the COVID-19 pandemic bears no resemblance to the media situation during previous pandemics. The internet has become a major resource in information transfer, and traditional means of communication are digitized and accessible online to the public at large. This access to the news *via* various platforms (e.g., live broadcasts, recordings and more) has increased the volume and repercussions of messages in the public sphere.

According to Dr. Glen Nowak, professor of advertising and public relations at the University of Georgia, in a news report by Elizabeth Dohms-Harter, media coverage of the COVID-19 crisis differs tremendously from coverage of the H1N1 crisis in 2009: “What's compounding coverage now is the reality that there are many more news outlets than there were in 2009, and more platforms such as Twitter and Instagram on which to spread that information. In 2009, Fox News wasn't as large, and outlets such as Stat and Vox didn't exist yet…. And as for social media, the CDC didn't even have a Twitter account in 2009. That didn't happen until May 2010” (1).

According to the professional literature, the media fulfill two roles during an epidemiological crisis. The first is to convince the public to listen to and comply with the guidelines, that is, to change the public's behavior (2). Research indicates that in order to change behavior, the media emphasize the urgency and gravity of the illness (3), thus influencing the public's perception of risk. Studies conducted during the COVID-19 pandemic indicate that media coverage has had an impact on two types of behavior (2). The first type is related to the issue of mobility. The message to the public during the lockdowns was to stay at home (2, 4, 5). For example, Chemli et al. found that “media have preeminent control on accentuating potential travelers' awareness during a crisis as the primary source of information” (6).

The second type of behavior affected by media coverage is related to vaccination compliance. Indeed, the media's second role during an epidemiological crisis is to provide information to the public. According to Matlis and Sonenshein (7), in order for people to make sense of potential threats during crises, they require a flow of timely and accurate information. This type of information flow can serve to increase people's trust in health organizations.

Many studies in the research literature have focused on how information on the pandemic is framed. Most studies examining these framing methods are conceptual in nature, with almost no empirical studies that investigate the effectiveness of these methods during the pandemic (8). Entman (9) claims that framing involves “selecting and highlighting some facets of events or issues and making connections among them so as to promote a particular interpretation, evaluation, and/or solution”. Neuman et al. (10) further explain that news frames are “conceptual tools which media and individuals rely on to convey, interpret and evaluate information”. Framing theory in the field of disaster communication suggests that the public's reaction to a disaster is not entirely determined by the type or magnitude of the disaster. Rather, it is heavily influenced by how the public interprets the disaster, which in turn is influenced by public relations and media framing (11).

Quite a few studies have examined the social construction of epidemics while focusing specifically on framing. For example some (12) identified the frames used in the Hong Kong media in covering the SARS crisis, including a frame that focused on governmental failures and the political implications of the outbreak. Others applied generic frames, such as attribution of responsibility, conflict, economic consequences, and human interest (13–15). Shih et al. (16) developed framing typologies specific to epidemic coverage (uncertainty, action, reassurance, conflict, and new evidence). In examining how *The New York Times* framed three health epidemics (mad cow disease, West Nile virus and avian flu), they discovered that the newspaper consistently employed two frames across the diseases: the action frame, which emphasized action taken to prevent the disease, and the consequence frame, which focused on disease consequences (e.g., victims, cost, social impact) (16).

Beyond the issue of framing information and motivating the public to respond/comply, as outlined above, very few studies examining the role of the media during the COVID-19 crisis have considered the following more fundamental questions: What type of information does the public obtain from the major news channels? Is this information accurate? Does this information reveal topics that are the subject of scientific controversy? Has the information undergone criticism (cross-checking of sources)? What is the role of the news media as the gatekeeper of democracy during emergencies such as epidemiological crises or pandemics?

Research studies in the field of health communications indicate that during routine times as well as during pandemics the traditional media tend to seek out sensations and dramatize the facts (17, 18). For example, Bomlitz and Brezis (19) investigated the relationship between causes of death in the US in 2003 and media coverage. Using the LexisNexis database, they counted US mass media reports on emerging and chronic health hazards (Severe Acute Respiratory Syndrome [SARS], bioterrorism, West Nile Fever, AIDS, smoking and physical inactivity) for 2003. Their findings showed that the number of media reports were inversely related to the actual number of deaths from the health risks evaluated. In other words, fewer than a dozen people were killed by SARS and bioterrorism in 2003, but together these hazards generated over 1,00,000 media reports, far beyond the number of reports on smoking and physical inactivity, which were responsible for the deaths of more than one million Americans. Moreover, during the H1N1 epidemic, researchers claimed that the media had unnecessarily sensationalized and hyped the threat posed by the virus in the interests of ratings and revenue (20, 21).

In addition to this media tendency to dramatize extraordinary health events and epidemics, the media also tend to use stories to make news items more personal. Indeed, this practice is considered a cornerstone in media coverage of health issues, particularly in the case of diseases and drugs (22). For example, during the mid-20th century polio epidemic, beyond providing scientific information, the American media offered sensational personal stories about people suffering from the disease (23). Similarly, in covering AIDS the Israeli media bombarded the public with narratives, myths, and compelling stories (24).

The research consensus is that the traditional media rely upon information from official organizations, professionals in senior positions or commercial bodies (25). Some studies contend that health journalists often rely upon scientists, medical professionals and academic centers as sources (26). Moreover, these journalists are more likely to cite mainstream scientific sources than those expressing conflicting opinions (27). Furthermore, journalists engaged in health communication often abuse their position by citing information without checking or examining it in depth, such that some of the information they convey may be misleading or only partially true and tainted by outside interests. Some studies indicate that health journalists tend to favor stories on new drugs and technologies. Indeed, the vast majority of medical and science news comes from short press releases that PR firms provide directly to journalists (28, 29). These press releases, which are often written by pharmaceutical companies, funding bodies or institutions that support clinical research, are designed to attract favorable media attention to newly published research results (30) and/or new drugs or technologies. In view of this, we must question the extent to which drug companies succeed in this goal.

In 2019 we conducted a study to examine what influences the ways in which the public reads and understands information. Our results showed that the media have trouble providing the public a critical examination of the methodology and research limitations on which public health policy is based (31). The aforementioned tendency on the part of journalists to omit such important information is characteristic of routine times as well as epidemiological crises (25).

During the COVID-19 pandemic, traditional media worldwide for the most part represented the official establishment line and gave almost no coverage to criticism of this official policy voiced by other experts (32). For example, examination of “elitist” media in the US, such as CNN and *The New York Times*, shows that these sources were almost always in line with the establishment and sometimes even reported facts that were not always accurate. Successive Gallup polls showed that the average Democrat believed that 50 percent of COVID infections resulted in hospitalizations, while the real number was <1% (33). In another example, Viswanath et al. (34) found that people who relied on mainstream print media like *The New York Times* or *The Washington Post* as their major source of COVID-19 news were more likely to get the vaccine. On the other hand, those who relied on Republican sources like Fox News as their major source of COVID-19 information were less likely to get the vaccine.

In December 2020 the International Press Institute (IPI) published a report summarizing the impact of COVID-19 on the press and on media freedom in the EU. The report analyzed the main types of violations and attacks monitored during the previous 10 months, documented key trends observed during that time, and assessed their impact on freedom of the press in Europe. According to the report, governments justified their actions because they gave government bodies more freedom to tackle the pandemic. Yet at the same time, these steps also undermined journalists' ability to obtain timely information about the rapidly changing health crisis. Indeed, numerous independent journalists complained that their requests for information from public institutions were being completely disregarded (35).

The IPI report analyzes the direct relationship between how governments managed the COVID-19 crisis and their attitude to the media, and primarily to journalists who were critical of them. Similarly, the current article seeks to examine the relationship between Israeli policy regarding the vaccination issue and the media. Studies conducted in Israel indicate that during the initial period, the COVID-19 crisis was overseen by only a handful of experts, political considerations were mixed in, and the opinions of experts who criticized government policies such as lockdowns were overlooked. This disregard for opposing expert opinions continued at the outset of the vaccination campaign in Israel (32, 36, 37).

In December 2020 the Israeli government announced plans to import the Pfizer pharmaceutical company's coronavirus vaccine. This announcement came after the FDA granted the Pfizer vaccine emergency authorization as an experimental drug (IND) until February 2023, when clinical trials were scheduled to end. According to Pfizer CEO Albert Burla in an interview with NBC, the contract it signed with Israel turned Israel into an experimental laboratory for Pfizer and the rest of the world as well (38). Within less than a month, the Israel Ministry of Health (MOH) recommended vaccinating medical personnel and at-risk populations, and subsequently recommended vaccinating the entire population over the age of 16 (39). The MOH's advisory board later recommended vaccinating children ages 5–11 (40, 41). Note that parts of this contract were and still are concealed from the public (42). Experts who criticized the government's vaccination policy were deemed “anti-vaxxers” and “Corona deniers” (32). Some of these critics even received letters of reprimand from the Ministry of Health (43).

The above discussion leads to the following question: How did the media in Israel cover the vaccination campaign? Reports on social media and esoteric items in the press indicate that the major media in Israel gave almost no platform to voices outside the establishment critical of the vaccination policy (44). Yet to the best of our knowledge, no empirical research has been conducted to corroborate this assumption. Moreover, as noted above, most studies examining the media and the pandemic focused on how the pandemic was framed and not on media framing of the vaccination campaign, as this study seeks to do. In addition, we are not aware of any empirical research that examined news coverage of the vaccination issue while analyzing the official government vaccination campaign.

This study seeks to examine these three issues by focusing on how Israel's two main television news channels (Channel 12 and Channel 13) covered the vaccination campaign compared to how the Ministry of Health ran the campaign.

## Methods

### Study Design

This qualitative study is based on triangulation of online content analyses from three different sources: advertising campaigns, social media posts and reports on television news channels (45). Furthermore, the study maps different media strategies using infographic charts. During the past two decades, the use of infographics has become more prevalent in qualitative research. This method has been found effective in depicting data in a clear, visual and succinct form (46).

### Study Sample

#### Television News Channels

The research sample included 252 reports from the newsrooms of Channel 13 and Channel N12, Israel's two leading news channels: 151 reports from Channel 13 and 101 reports from Channel N12, all broadcast between December 1, 2020, and November 30, 2021. The reports were sampled by the directed sampling method (47). This entailed examining the archives of Channels 12 (*n* = 305) and 13 (*n* = 524), locating reports from both channels (*n* = 829) that included the word “COVID- 19”^1^ and watching the news videoclips of these reports. Finding the reports for the research sample entailed searching the N12 news website using the keywords “corona” and “vaccinations” and screening all the results that included videoclips from the main news broadcast. On the Channel 13 news website, the search was conducted by filtering reports with videoclips under the main newscast tab that referred to corona and vaccinations.

Inclusion criteria were as follows: only content articles and not news items, only reports that appeared on the main evening news broadcasts, articles that dealt with the vaccination campaign in Israel only, reports that included interviews with experts and citizens about the vaccination campaign, and reports that included morbidity and mortality data in the context of calling upon the public to be vaccinated. Exclusion criteria were as follows: other items that were only broadcast on the news on these channels but were not included in the written reports on the websites.

**Figure 1:**
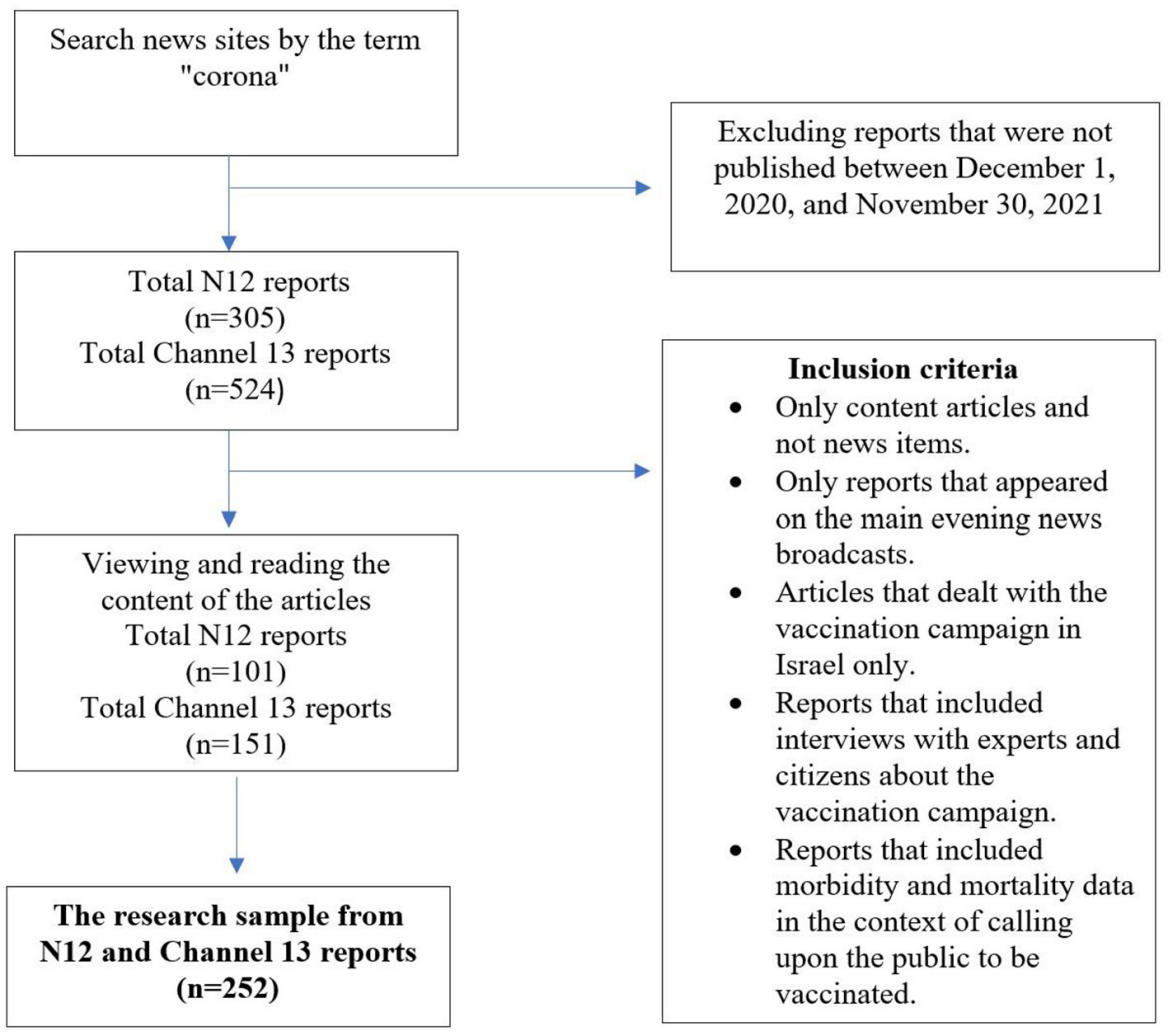
Sampling flowchart.

The following flowchart illustrates the sampling method (Figure 1).

#### Ministry of Health Facebook Page and the Israel Government Advertising Agency (LAPAM) Campaign

In addition to sampling reports from the news channel websites, the research also sampled posts from the Ministry of Health Facebook page and advertising campaigns from the Facebook page of the Israel Government Advertising Agency (LAPAM), which constructs advertising campaigns for the MOH. A total of 113 items focusing on the coronavirus and on promoting vaccinations among the Israeli population were sampled between December 2020 and November 2021. The sample was divided between 77 posts on the MOH Facebook page and 36 LAPAM campaigns. Of these, a total of 101 items did not appear on both sites.

### Validity and Reliability

Throughout the research, all information relevant to the study was stored, from the raw data collection stage through the analysis stage and up to the stage of writing up the findings and drawing conclusions (48). In addition, the researchers consulted each other regarding the data so as to facilitate discussion of the findings from the initial collection through the interpretation stage (49). The analysis included watching the videoclips of the news and reading the posts and the advertising materials. The two researchers examined the materials separately. In accordance with the inter-rater reliability method, the three researchers discussed how to formulate the criteria and select the themes and quotations (50).

### Data Analysis

In the first *stage* of the research, we built mapping tables that included the following data: date of the post or news report, the item's target audience, the message transmitted, whether the item was related to vaccination, and how it encouraged citizens to get vaccinated. We build three separate tables, one for each source (news channels, MOH Facebook page, LAPAM Facebook page).

The second *research stage* entailed conducting separate online content analyses (51) of the messages transmitted in the posts, the advertising campaigns and the news reports. The content analysis included coding major concepts and identifying primary and secondary themes. The purpose of this content analysis was to discover the framing methods that the MOH and the newscasts used to convey messages about COVID-19 vaccinations.

In the *third research stage*, we discussed the common themes emerging from all the data collected from the three sources until achieving inter-rater agreement. Note that practically no differences were found between the themes emerging from the MOH data and those emerging from the newscasts.

In the *fourth research stage*, we triangulated the findings by means of an integrated table that depicted the strategies/criteria shared by the newscasts and the MOH posts and campaigns: positive framing fear appeal, attribution of responsibility frame, and vaccination status.

In the fifth *research stage*, we chose quotations that were relevant to the themes/framing methods identified in the research.

The sixth *research stage* entailed building an Excel table that depicted the number of times the MOH/LAPAM used these strategies in a given month compared to the number of times the newscasts used these strategies, during the months for which data were collected (December 2020 through November 2021).

In the seventh *research stage*, we used Adobe Illustrator CC2022 to construct infographics for each framing method separately, while comparing the MOH to the news channels for all the months in the sample.

The eighth *research stage* entailed analyzing the trends depicted in each graph for each month of the research, while inserting relevant quotations to support the findings.

In the ninth *research stage*, we added a word cloud constructed by identifying names and labels attached to citizens and experts who criticized the vaccination policy or chose not to be vaccinated for various reasons. See the Results section for more details on word cloud representations.

## Results

### Use of Positive Framing: Newscasts vs. Ministry of Health

**Figure 2:**
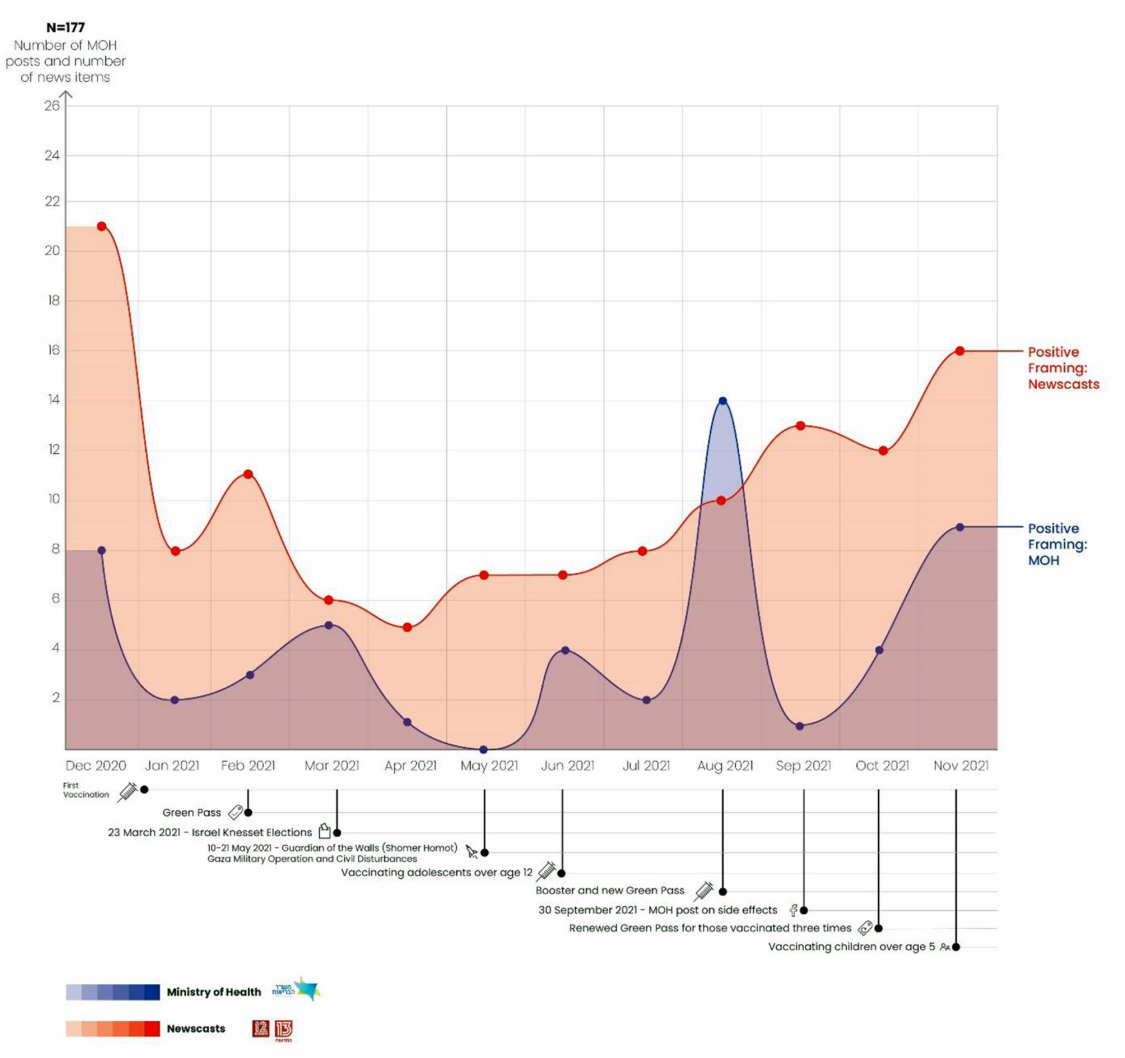
Use of positive framing: Newscasts vs. Ministry of Health.

A total of 177 news reports and posts on the MOH Facebook page were identified as using positive framing. As can be seen in Figure 2, at the beginning of the vaccination campaign in December 2020, a large number of posts and reports both on the news and on the MOH Facebook page used positive framing by depicting the vaccination as the solution for eradicating the epidemic. For example, in a report broadcast on the N12 news about the opening of a new vaccination site at Ichilov Hospital, Dr. Esther Saiag, Deputy Director of the hospital's Medical Systems Operations, described the launching of the vaccination campaign: “Remember? Corona patients, despair, sorrow, depression? Here we are moving from darkness to light. We are closing the cycle in the most graphic and optimistic way possible” (52).

One of the MOH's first posts about the launching of the vaccination campaign stated: “The vaccination was approved by the FDA and tested on tens of thousands of people. The vaccination campaign in Israel is starting now and will continue for several months. Only when most of the population gets vaccinated can we hope to return to normal… The vaccination is the way to defeat the epidemic” (53).

The number of reports and posts began to decline in January 2021. By February the number began rising again, in advance of implementation of Israel's vaccine certificate policy known as the Green Pass Policy. At this point, both the news and the MOH continued with their positive framing of the COVID-19 vaccination, together with the Green Pass and its advantages. For example, Noga Nir Neeman of the Channel 13 news team noted that the Green Pass constitutes a passport for citizens to return to normal: “Yes. It sounds complicated and will probably involve quite a few glitches. But at least the complications mark our return to normal” (54). The following MOH post is in a similar vein: “Have you received your second dose of the COVID-19 vaccine? Great! You are entitled to a vaccination certificate that exempts you from isolation” (53).

Between March and May 2021, two significant and dramatic events took place in Israel: General elections for a new legislature were held in March. Then in May Israel launched a military operation in Gaza known as Guardian of the Walls (Shomer Homot), and civil disturbances broke out in many Israeli cities. During this period, the media and the government were preoccupied with these events, such that there was a decline in the number of MOH posts that used positive framing for the vaccinations. The newscasts revealed minor fluctuations in the number of reports using positive framing, with a slight decline in April followed by an increasing trend in the number of these reports.

In June 2021 when the vaccination was approved for adolescents over the age of 12, the graph shows another sharp rise in positive framing. It is important to note that the newscasts mentioned vaccination of those over age 12 even before the MOH approved this step. These news reports emphasized that the vaccination would help curb the spread of the virus and protect the older at-risk population group. In a March news report about vaccinating children over the age of 12, Prof. Gabi Barbash made the following statement: “Why should adolescents be vaccinated? Because we know that if we do not put a stop to this cycle of infection and mutation, we will never be able to rid ourselves of this virus completely. Remember that we give vaccinations to prevent childhood diseases. For example, children are given the triple vaccine against measles, mumps, and rubella, usually without any ill effects. We vaccinate them because we do not want the children to get sick but also to protect the adults. So, generally speaking, we want to create a situation in which enough people are vaccinated so that this disease is under control” (55).

From June through November 2021, the use of the positive framing strategy on the news continued to rise. Practically no mention was made of side effects, with the exception of those that were temporary. For example, a Channel 13 news report quoted Health Minister Nitzan Horowitz: “The results of the most recent studies on the effectiveness of the booster are unequivocal. The vaccination is safe and extremely effective in preventing infection. Moreover, it dramatically reduces the chances of serious and life-threatening illness.” The report went on to say: “To date about two million Israelis have had the third vaccination. The hope is that offering the booster to the entire population will contain the fourth wave” (56).

In August, in advance of the booster and the Green Pass, the MOH's positive framing increased significantly, as can be seen in the following quotation: “With the Green Pass, doors are opening for you: You can go out with friends, to the gym, to performances, to the movies…. Because with the Green Pass life goes on, so it pays to get vaccinated” (53).

On September 30, 2021, the MOH posted an item about vaccination side effects that targeted the general public: “Let's talk about reported side effects after getting the COVID-19 vaccination. The vaccinations are safe and effective. The MOH will continue to provide regular reports on side effects and will cross check this information with data collected in other countries across the globe and with clinical trials conducted on the vaccinations…” (53).

This post received 28,000 reactions from the public, most of which mentioned reported side effects not mentioned by the MOH in its post. The MOH responded to the public by deleting some of these reactions, claiming they were violent and contained threats against MOH personnel. The public continued to claim that the MOH was not providing a fully transparent picture of the side effects, as seen in the following item in the press: “When the MOH published a post on the side effects of the COVID-19 vaccination, it did not expect such a storm in response. Comments that expressed criticism or contradicted the Ministry's decisive opinion by reporting side effects should not be deleted. Certainly not from a post that is supposed to invite discussion” (57). At the same time this MOH post on side effects appeared, the news continued its positive framing of the booster. Indeed, prior to the campaign to vaccinate children launched in November 2021, an increase can be seen in the number of reports and posts that framed the vaccination positively.

### Use of the Fear Appeal Strategy on the News vs. the MOH's Positive Framing Strategy

**Figure 3:**
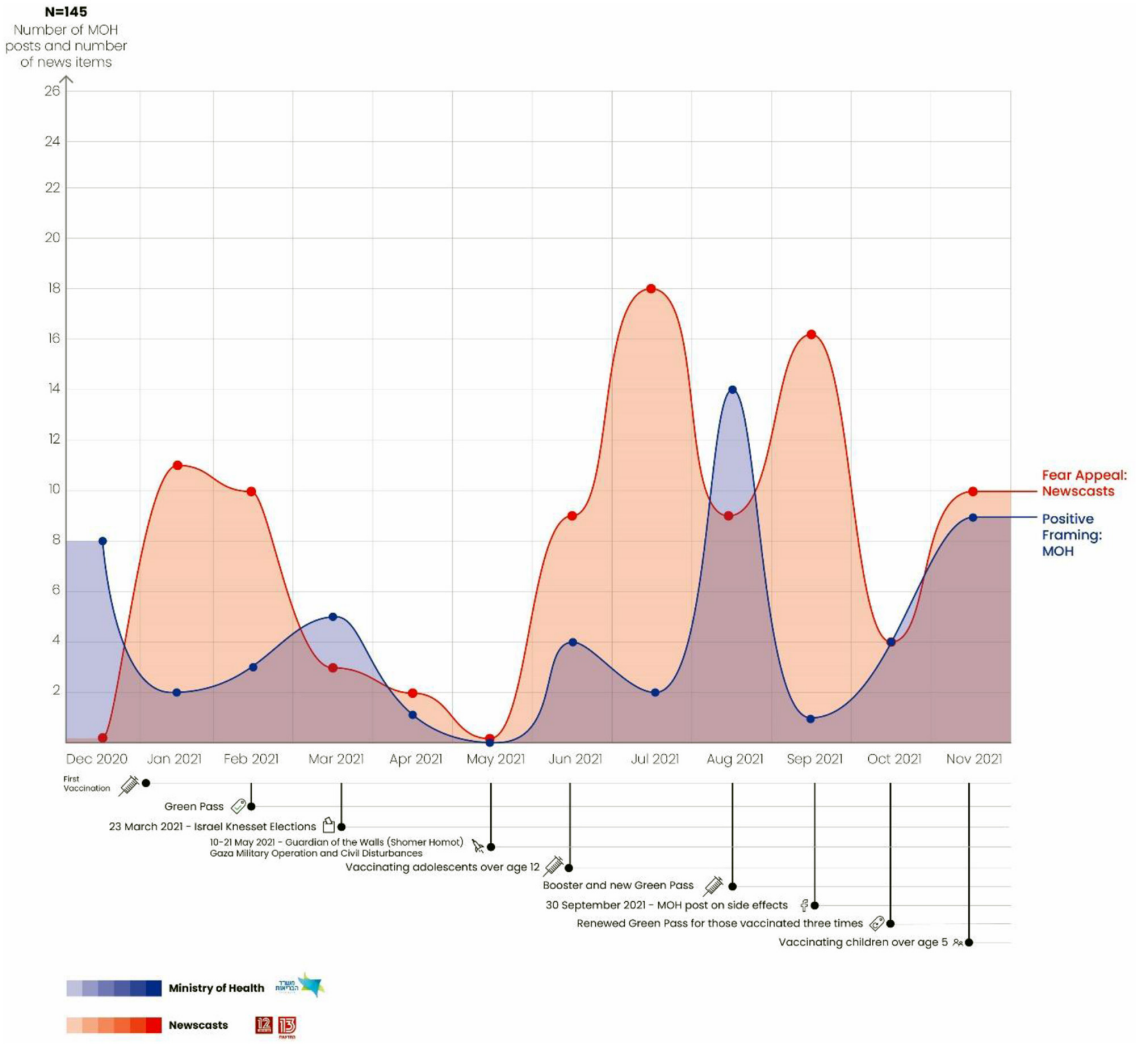
Use of fear appeal strategy on the news vs. MOH's use of positive framing strategy.

A total of 145 news reports were identified as using the fear appeal strategy in parallel to MOH posts that used the positive framing strategy. The graph in Figure 3 shows that when the MOH began its first vaccination campaign in December 2020 it used positive framing to motivate the public to get vaccinated and be eligible to receive a Green Pass. Simultaneous to the MOH vaccination campaign, the graph shows a parallel rise in the number of news reports using the fear appeal strategy. For example, Prof. Barbash stated on the Channel 12 news that the vaccination is intended to protect people of all ages from serious illness: “Getting this disease is lousy at any age” (58).

From January through March, in advance of the Green Pass, the trend toward positive framing continued in the MOH posts, while until February the media continued to use the fear appeal strategy. For example, a report on the Channel 13 news that described the difficult and overcrowded conditions in the COVID-19 ward at the Hadassah Ein Kerem Medical Center demonstrates the use of the fear appeal strategy: “The fifth COVID-19 ward opened today at the Hadassah Ein Kerem Medical Center. Within the first hour, Dvori Duchan, Director of Nursing, admitted one patient after another after another. She knows that by tomorrow the ward will be completely full, with patients occupying all thirty beds” (59).

As can be seen in the graph in Figure 3, between February and May the number of MOH posts declined, as did the number of news reports. Note that the Israeli elections took place in March and the military operation and civilian disturbances occurred in May, so that the majority of the media discourse revolved around these events. At the same time, most of the COVID-19 restrictions were lifted, which explains the decline in the number of reports using the fear appeal strategy.

By June 2021, after the disturbances came to an end and the new Israeli government was formed, the use of the fear appeal strategy on the news increased, as did the MOH's use of positive framing. From July through September, there was a sharp rise in the number of news reports using fear appeal, along with a decrease in the number of positively framed MOH posts. That is, the graph depicts a mirror image, such that when one curve shows an increase the other shows a decrease. Note that during those months, the campaign to vaccinate adolescents over the age of 12 began, as did the booster campaign. When the post about side effects was published on the MOH Facebook page on September 30, the MOH adopted a positive framing strategy to emphasize the advantages of the vaccination, as can be seen in the following quote from the MOH: “The new Green Pass is underway. You can live a normal life, the economy is open and fully operating, including the school system” (53). At the same time, the use of the fear appeal strategy on the news decreased. In advance of the decision to give the vaccination to children, both graphs exhibited an increase in the number of reports using these two strategies.

### Use of Fear Appeal Strategy: Newscasts vs. Ministry of Health

**Figure 4:**
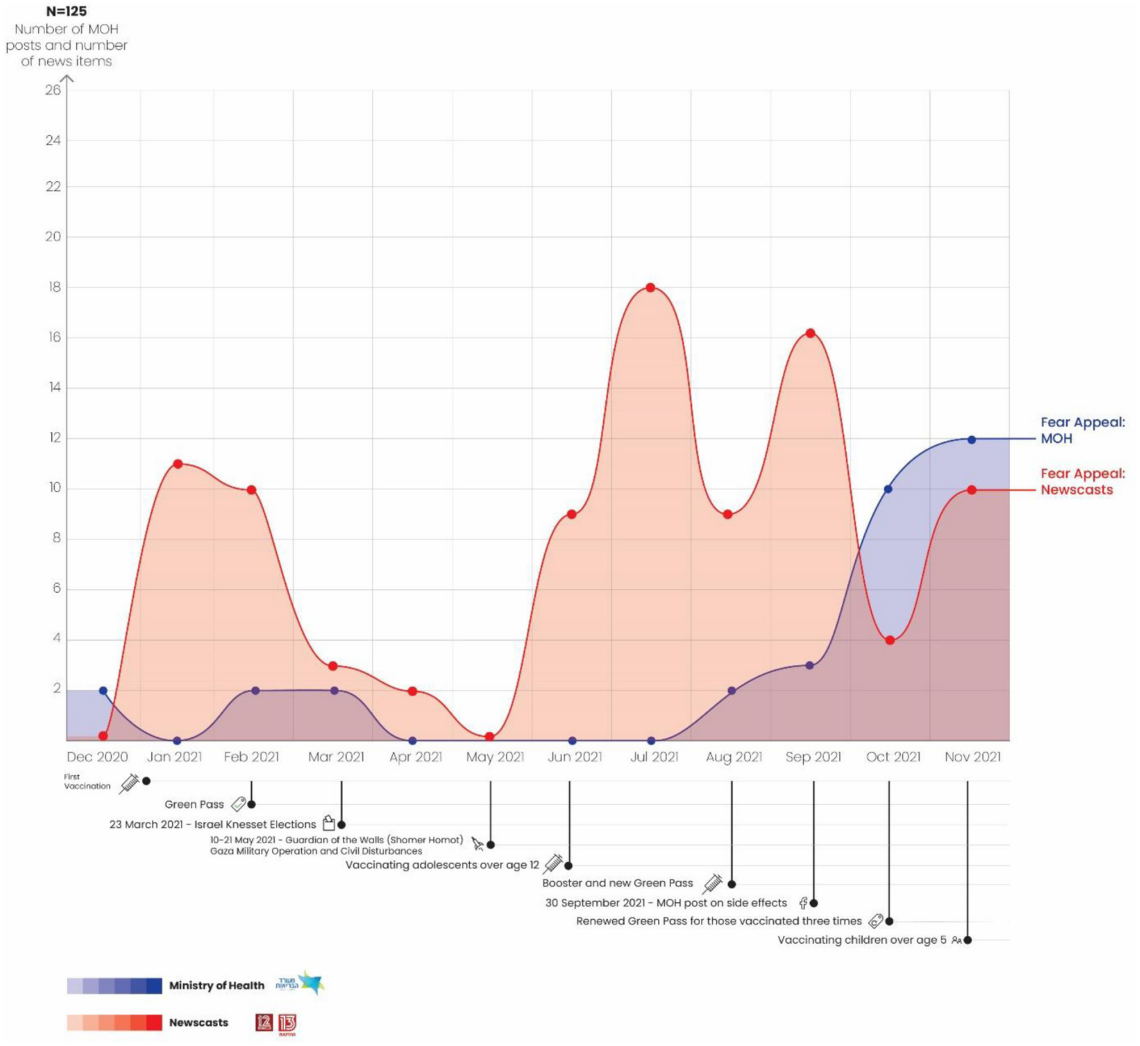
Use of fear appeal strategy: Newscasts vs. Ministry of Health.

A total of 125 news reports and posts on the MOH Facebook page were identified as using the fear appeal strategy. As can be seen in the graph in Figure 4, from the beginning of the vaccination campaign in December 2020 through July 2021, the MOH made almost no use of the fear appeal strategy. From August, an increasing trend toward using the fear appeal strategy is apparent in the MOH posts, including the booster vaccination, cancellation of the Green Pass and vaccination of children over the age of 5. At the same time, the news reports also demonstrate a sharp rise in the use of the fear appeal strategy from January through February, in advance of the Green Pass (with a slight drop in February), followed by a decline through May. Note that both the Israeli elections and the military operation and civilian disturbances took place during this period, so that the majority of the media discourse did not focus on the coronavirus crisis.

From June through July, in advance of the vaccination of adolescents and the booster, a significant and ongoing rise in the use of the fear appeal strategy on the news is apparent. This can be seen in the following quotation from an N12 newscast that focused on the rise in the number of confirmed COVID-19 cases and the third vaccination being offered to those with compromised immune systems: “Even when the numbers are low, the coronavirus continues to claim victims, like Yitzhak and Rachel Naeh, who died within 4 days of one other.” The report continued by interviewing the victims' son, who stated that his parents had received both doses of the vaccination and were about to get the booster: “They were vaccinated. Even before the vaccinations were available, they looked after themselves. We behaved by the book this whole year. We isolated them, saw to their needs, all by the book. And now we've become complacent. Not we as a family, but we as citizens” (60).

In August, the graph for news broadcasts exhibits a mild drop in the use of the fear appeal strategy. After the MOH published its post about side effects on the MOH Facebook page on September 30, the graph shows a significant rise in the MOH's use of the fear appeal strategy. Indeed, the MOH sought to point out the negative consequences if citizens do not get the booster and do not vaccinate their children. At this stage the MOH's fear appeal strategy found expression in a series of posts featuring people who had recovered from serious cases of COVID-19. These people told about their experiences with the disease and conveyed the importance of the vaccination: “‘Here's how bad it was. After going to the kitchen you find yourself panting for the next 20 min. ' David Ganish believed the fake news and didn't get vaccinated. He became infected with the coronavirus and now is undergoing lung rehabilitation that is expected to last at least 3 months. He has one message for you: Get vaccinated!” (53).

During this period the graph for the news shows a similar rising trend in fear appeal. In October, however, this general fear appeal trend is replaced by an additional strategy we refer to as attribution of responsibility, as discussed below. In other words, the general strategy of fear appeal took on an additional aspect in the form of attributing responsibility to the public in advance of the vaccinations being offered to children from the age of five.

### Use of Positive Framing Strategy on the News vs. MOH Use of Fear Appeal Strategy

**Figure 5:**
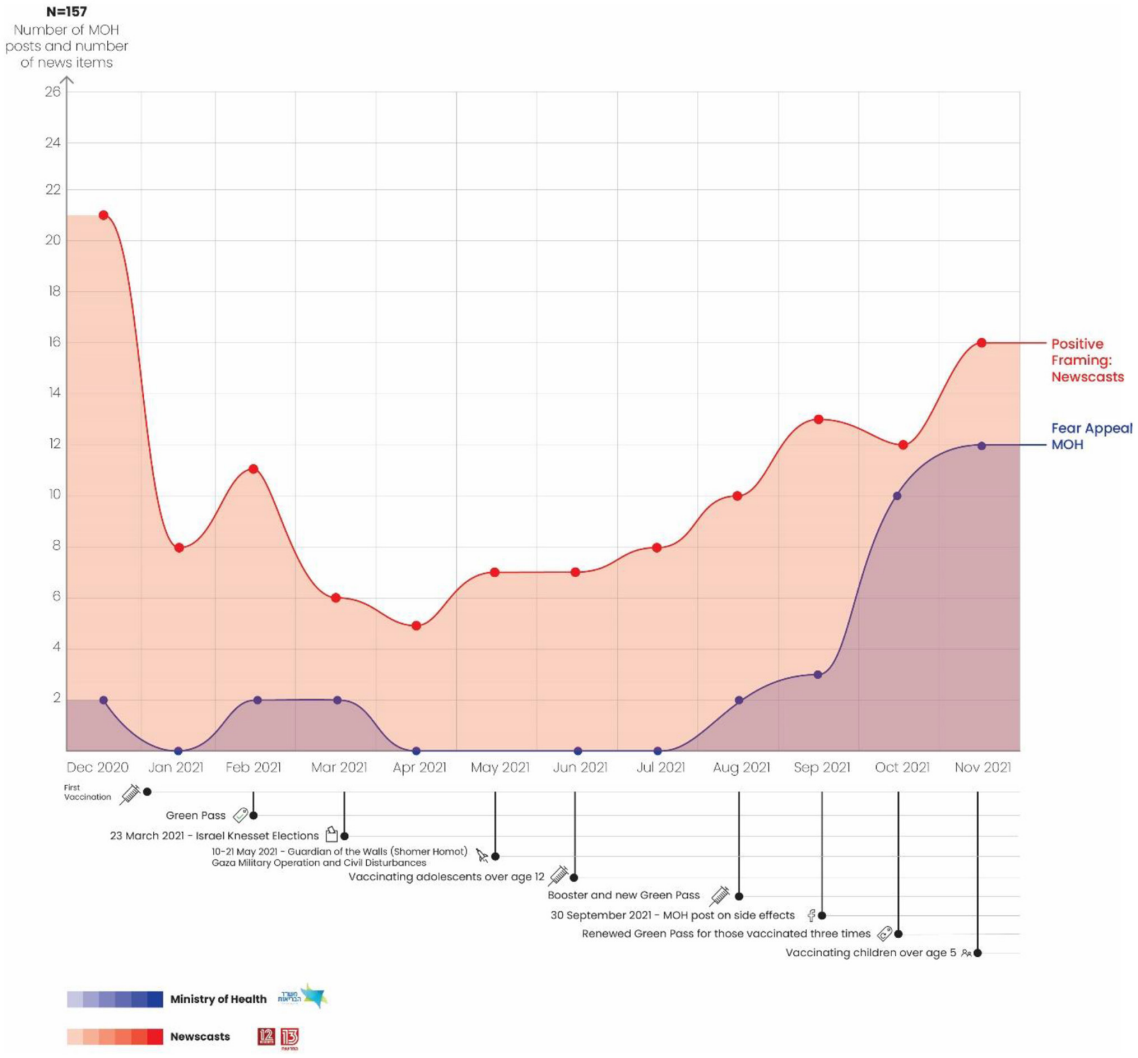
Use of positive framing strategy on the news vs. MOH use of fear appeal strategy.

A total of 157 news reports were identified as using the positive framing strategy in parallel to MOH posts that used the fear appeal strategy. Figure 5 shows a generally consistent trend both for the MOH and for the news during the entire period (December 2020 through November 2021), such that when positive framing rises on the news there is a parallel rise in fear appeal strategy for the MOH. These two significant increases—in the use of positive framing on the news prior to the first vaccination and the use of fear appeal by the MOH in laying the groundwork for vaccinating children—provide a complementary picture of the use of these two contrasting strategies from August through November 2021).

### Use of Attribution of Responsibility Strategy and Mentioning Vaccination Status on the News vs. MOH Use of Attribution of Responsibility Strategy

**Figure 6:**
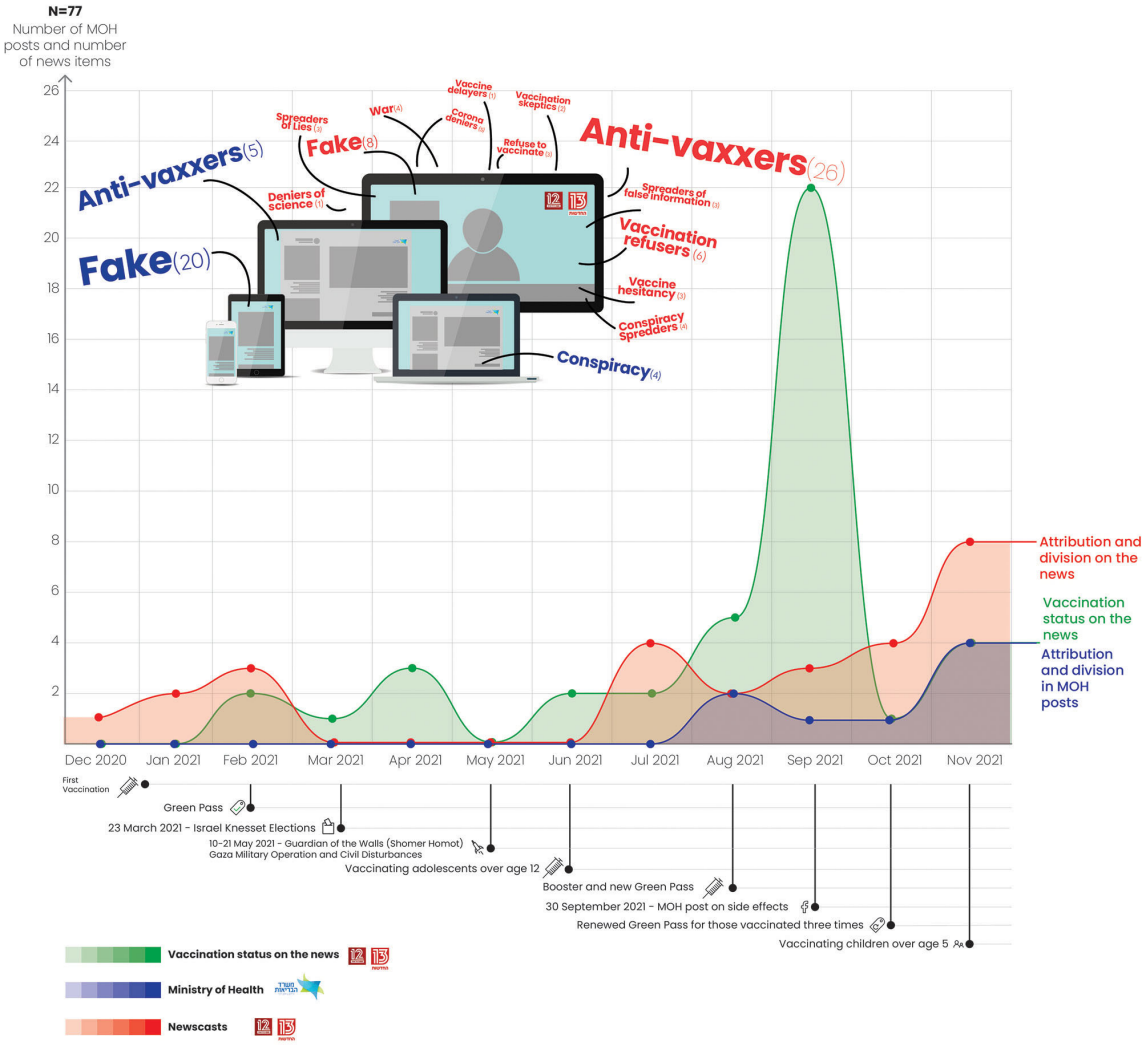
Use of attribution of responsibility strategy: Newscasts vs. Ministry of Health Plus Word Cloud depicting vaccination status.

A total of 77 news reports and posts on the MOH Facebook page were identified as using the attribution of responsibility strategy. This strategy usually entails assigning negative tags to people who decided for various reasons not to be vaccinated or to professionals who opposed the vaccination policy. As can be seen in Figure 6, while the news adopted this strategy, up until July 2021 the MOH did not use it at all. The following quotation from a Channel 13 newscast illustrates this attitude toward people who did not get vaccinated: “Not only is the vaccination campaign expanding. Anti-vaxxers are also spreading more outrageous fake announcements that make false claims about the safety and effectiveness of the vaccines. Particularly outrageous is that some vaccination opponents have posted notices on Facebook calling on people to make vaccination appointments and then cancel them at the last minute so that the unused vaccines will have to be discarded” (61).

Starting in August, the MOH began using the attribution of responsibility strategy in the context of the booster vaccination, the new Green Pass and vaccination of children from the age of five. For example, the following MOH post is made up of a collection of public slogans stamped with the words “Warning—Fake News” in red: “Here's who is behind these dangerous statements: a doctor whose license has been revoked and a group advocating an alarming ideology that rejects scientific consensus. If it was up to them, our children would not be vaccinated against polio, hepatitis, measles, tetanus and whooping cough. You would not take their advice on how to treat a headache, so why listen to them when it comes to the health of your children?” (53).

The news also used another strategy to complement the attribution of responsibility strategy. We refer to this strategy as publicizing vaccination status while differentiating between those who received three doses of the vaccination and those who received one or two doses but did not get the booster. Both the MOH and the media referred to these individuals as being “unvaccinated” or “not fully vaccinated” or whose “vaccination expired”. The discourse on vaccination status from the beginning of February through April was intended to underscore the differential diagnosis for obtaining the first Green Pass. This diagnosis distinguished between those who were vaccinated with one dose, those vaccinated with two doses and unvaccinated individuals. As in the other graphs, the data in Figure 6 also reflect the Israeli elections in March and the military operations and civil disturbances in May, which deflected the public discourse from the coronavirus situation. From June and in anticipation of the vaccination of adolescents over the age of 12 and the booster shot, the use of the vaccination status strategy showed a rising trend. This trend intensified at the beginning of the booster campaign (August 21) through September 21.

In Figure 6 we also added a word cloud constructed from MOH posts and news reports (*N* = 55) that made references to citizens who were not vaccinated or not fully vaccinated or to professionals who opposed or criticized the MOH vaccination policy. The word cloud shows the prevalent use of negative terms such as “anti-vaxxers”, “corona deniers”, “conspiracy spreaders”, “spreaders of fake news” and more. The figure shows congruence between the use of these words and the use of the attribution of responsibility strategy.

## Discussion

### Congruence Between the Ministry of Health and the News Channels in Framing the Vaccination Issue

The research findings reveal congruence between the way the MOH framed its vaccination campaign and news coverage of the vaccination issue. Framing is defined as the selection of “some aspects of a perceived reality” to enhance their salience “in such a way as to promote a particular problem definition, causal interpretation, moral evaluation, and/or treatment recommendation” (62). As noted in the literature, framing is important in that news frames serve as “conceptual tools which media and individuals rely on to convey, interpret and evaluate information” (10). The way an issue is framed can change the reader's perception. In framing, the same information serves as a base and the actual facts are not altered. Rather, in covering a story the media choose certain words and images to convey the frame (63). In the context of epidemiological crises that involve politics, a frame defines how an element of rhetoric is packaged so as to encourage certain interpretations and discourage others.

As noted in the Introduction, many studies have examined media framing of the pandemic in reference to issues of urgency and severity along with social and economic consequences (12, 13). Yet almost no research has examined how the media framed the vaccination campaign, as this study has done. Our examination of the strategies the MOH and the Channel 12 and 13 newscasts used to frame the vaccination campaign yielded three primary framing strategies: positive framing, fear appeal and attribution of responsibility.

#### Positive Framing: MOH and News Coverage

Both the MOH and the news coverage of the vaccination campaign framed the vaccinations positively as the ultimate solution for containing the epidemic. During the initial weeks of news coverage, the television channels emphasized the advantages of the vaccinations, including their ability to contain the epidemic and to protect people from infection. They did so despite awareness that the vaccine had been developed rapidly using a technology that had previously been used only in Pfizer's clinical trials (64–66), such that its effectiveness could not be assessed. In addition, already in a Pfizer trial and at the outset of the vaccination campaign in Israel, it became clear that the Pfizer vaccine does not prevent contagion or stop the virus from spreading in the population (67).

The MOH expanded its vaccination campaign to include all age groups, not just the older at-risk population. In doing so, it emphasized the vaccine's advantages, stressing again and again that the vaccine is safe and effective based on cost-benefit calculations and on public health perspectives (68) and that its advantages outweigh its disadvantages (69, 70).

Concurrently, the newscasts promoted the narrative that the vaccination is effective for all groups in the population, without providing a forum for other experts who opposed the vaccination policy to voice their opinion. Among the reasons for these experts' opposition were that the government's sweeping recommendations to vaccinate the entire population failed to note the absence of long-term and thorough research to follow up the vaccine's side effects. They also expressed concerns about side effects that had already appeared (71), such as myocarditis in young people (72) and coagulation problems (73). In objecting to universal vaccination, these opponents claimed that at-risk populations and people with underlying conditions should be vaccinated, while the rest of the population should be given the right to make autonomous decisions rather than subjected to direct or indirect coercion (e.g., by the Green Pass that confers privileges on those who have been vaccinated) (74). Moreover, they argued against emergency vaccination of children (75), both because COVID-19 is not dangerous for children and due to concerns about vaccine safety (71).

The findings of this study indicate that the MOH vaccination campaign did not reveal the disadvantages of the vaccination, with the exception of one post on 30 September 2021 that described selective side effects. This post aroused anger among many people, who claimed that the MOH had not been fully transparent in revealing the vaccine's side effect. Indeed, thousands of citizens posted reports about side effects not mentioned in the MOH post. In response, the MOH chose to delete some of these responses, claiming that the deleted posts had used violent and threatening language. As a result of this deletion, the MOH received additional reports from citizens about the vaccination's side effects. This strategy of disregarding and deleting responses from the public led to what is known in the literature as a boomerang effect (76). That is, rather than showing the public that the MOH was being transparent, the post aroused public rage.

Similarly, on September 10, 2021, an ABC affiliate in Detroit solicited stories on its Facebook page about unvaccinated people who had died from COVID. The response was not what the network had wanted or expected: More than 2,30,000 people responded with messages containing heartbreaking stories of injuries and deaths from vaccines. Within 10 days, readers had shared this post more than two hundred thousand times (77).

Concurrently, the findings show that the newscasts on Israel's Channel 12 and Channel 13 continued to echo the advantages of the vaccination, almost without giving a voice to citizens' reports or scientific articles on side effects. This media approach of almost exclusively singing the praises of vaccination to a large extent reflects the pharmaceutical industry's narrative that a particular drug/vaccine meets the needs of humanity in coping with the pandemic. This drug narrative entails a story or script in which “problems” that arise are “solved” by drugs (78). This type of narrative is used to frame information about a drug and usually includes “environmental messages” that frequently identify “unmet needs”.

#### Fear Appeal: MOH and News Coverage

Alongside framing the COVID-19 vaccination as the ultimate solution, both the MOH and the newscasts used the fear appeal strategy. This strategy involves conveying persuasive messages that seek to arouse fear through threats of impending danger or harm, thus changing the way people behave (79). Throughout the vaccination campaign, the newscasts stressed the high morbidity and mortality rates from COVID-19 (32). The findings of this study show that when positive framing of vaccinations on the news rises, a parallel rise in the MOH's use of the fear appeal strategy is also apparent. Moreover, the two significant rises in the use of the positive framing strategy on the news and the use of fear appeal by the MOH formed complementary mirror images, as seen prior to the offering first vaccination and in preparation for the campaign to vaccinate children (August through November 2021).

During recent disease outbreaks, health organizations and the media appear to have reinforced their apocalyptic narrative by using strategies of intimidation to make the public comply with instructions and guidelines (80). For example, Bonneux and Van Damme (81), epidemiologists and scholars in the field of public health, referred to the HINI pandemic as an “iatrogenic pandemic of panic, an artifact caused by the treatment of the problem that is created by the way health officials and the media handled the health crisis”. In addition, Wagner-Egger et al. (21) found that many laypeople see the media as “fear mongering or as a puppet serving powerful interests”. Moreover, WHO Director General Margaret Chan and Assistant Director-General Keiji Fukuda have suggested that the media are a contributing cause of heightened risk perceptions among the public (82).

The use of the fear appeal strategy in Israel during the COVID-19 pandemic also emerged in other studies we conducted, for example in the language and tone politicians used to deliver information in the media (e.g., the use of war language to describe COVID-19 as a cruel enemy that needs to be defeated) (37). Yet we found that this type of apocalyptic pandemic narrative can be problematic in that the use of intimidation without the empowerment of individual self-efficacy contradicts the extended parallel process model (EPPM) (80). According to this model, in order for fear-based policies to be effective, policymakers “must induce a moderate level of fear alongside a higher level of self-efficacy and response efficacy. When the public's fear exceeds its sense of self-efficacy, the message becomes ineffective” (83).

#### Attribution of Responsibility: MOH and News Coverage

Our findings also show that both the MOH and the news coverage used the attribution of responsibility frame, which is defined as “a way of attributing responsibility for [a] cause or solution to either the government or to an individual or group” (15). In our results, this framing found expression in attributing responsibility for spreading the virus to what was referred to as “the group of unvaccinated people in Israel” (84). Even though studies had already indicated that both vaccinated and unvaccinated people were infected by the virus and infected others, this group was attributed primary responsibility for the failure to contain the pandemic. The news often distinguished between vaccinated and unvaccinated groups, though the definition of vaccination status varied. That is, someone in the unvaccinated group may have received both the first and second dose but was still assigned to the “unvaccinated” group on the news. This strategy was designed to show that people from the “unvaccinated” group who were hospitalized placed a burden on the entire medical system. It also attempted to create a rift between the two groups such that they would fight against each other.

This strategy of “divide (between the vaccinated and unvaccinated) and conquer (the vaccinated)” also targeted all those who opposed or criticized the generic vaccination policy in Israel. Some of the critics were renowned experts, including medical experts who, according to the research findings, were called “anti-vaxxers” and “corona deniers” even though their positions and/or actual behavior (e.g., media information regarding whether they themselves were vaccinated) did not justify this.

These attempts to ostracize and label anyone who criticized the official vaccination policy or refused to be vaccinated as an opponent or an outcast helped create a spiral of silence in society. The spiral of silence theory claims that when people notice that a group shares their personal opinion, they are more inclined to be confident and outward in expressing themselves. On the other hand, if people see that their opinion is not popular, they are more likely to be reserved and silent (85, 86). In Israel, this spiral of silence spilled over into many areas, for example among doctors who noted that the system was pressing them to “toe the line” and was intolerant of any expressions of hesitancy (87).

Furthermore, prior to the pandemic schisms in Israeli society revolved around religion (secular vs. religious), ethnic origin, nationality (Arabs vs. Jews) and socioeconomic status rather than around issues of health (88). The way the COVID-19 crisis was managed and constructed in the news generated a new social schism in Israeli society that had never existed before: between the vaccinated and the unvaccinated, a phenomenon that has also occurred all over the world. Until the COVID-19 crisis, vaccination was an issue decided upon by each citizen as a matter of respect for human dignity and liberty. The crisis, which led to new emergency regulations in the form of the Corona Bill, created a contrary movement toward coercion, such that anyone not complying or showing hesitancy became an outcast citizen. The news media did not criticize this shift away from respect for human dignity and liberty.

### The News as the MOH Watchdog

It is important to stress that this study examined newscasts only and not other media broadcasts on television, which may be interpreted as legitimate commentary on information. In other words, the news on television is not simply another television program. Rather, to a large extent citizens see the news as “reality” and as “the watchdog of democracy”, whose job it is to provide the public with complete and balanced information by investigating and cross-checking various sources. In today's changing media environment and against the background of global economic forces and wars over religion and culture, we must ask ourselves whether this watchdog metaphor still holds. According to Bourdiet, power relations range between autonomy and heteronomy (89). In what way do these relations find expression in media health reporting during the pandemic?

Dew (90) contends that the media generally adopt “a passive attitude to information produced and disseminated by established medical and public health institutions”. Yet according to Pfund and Hofstadter (27), in reporting controversies the traditional media have a tendency to refer to mainstream scientific sources rather than alternative opinions. In framing such controversies, the media rely on “the sound of science” rather than attempting to question or challenge them (90). Indeed, research shows that during routine times as well as during previous epidemiological crises, journalists tend to receive their information in the form of press releases from government ministries and from industry sources, without examining the reliability of this information. According to Park and Reber, these press releases may even influence the ways in which journalists formulate and frame health news (91). The power of these press releases emerges in a study that examined press releases and abstracts of scientific articles (30). The results show that nearly half of the items they examined included some degree of “spin” and that “spin” in press releases was associated with “spin” in article abstracts. Jackson (92) invented the term “churnalism” to depict this journalistic tendency to use press releases without examining them carefully.

The findings of the current study also point to the media's tendency to accept the MOH narrative, as seen in the congruence between the media's framing strategies and those of the ministry. This indicates that the newscasts chose to report the “news” according to the values adopted by the MOH. This congruence reflects what the literature refers to as news values, i.e., “criteria that influence the selection and presentation of events as published news, which help explain what makes something newsworthy” (93).

In Israel and in the rest of the world, the media's echoing of official messages helped determine the boundaries of the political discourse on the pandemic. These boundaries, also known as the Overton Window, helped define what was permitted and what was prohibited (94, 95). Throughout the entire research period, no news investigations were broadcast that attempted to answer fundamental questions regarding crony capitalism, a situation in which individuals and businesses with political connections and influence are favored. For example, the clandestine contract with Pfizer was never investigated. Moreover, no studies examined conflicts of interest in Israel or elsewhere in the world, for example pharmaceutical company contributions to organizations such as the FDA. In fact, 46 percent of the FDA's budget comes from “user fees” paid by the pharmaceutical industry (96). In Israel, no research examined the contributions made by pharmaceutical companies (including Pfizer) to the MOH, as reported in the MOH report on health donations (97). Nor have there been any studies investigating how the MOH finances its publication and media budgets. Despite the MOH's ongoing budgetary deficit, within a year it transferred two hundred million shekels to media and PR companies (98). Furthermore, no research has investigated economic conflicts of interest, such as the lack of competition between the vaccinations and other medications found effective in treating COVID-19, such as the scientific controversy surrounding the attempt to use hydroxychloroquine as a potential treatment (99–101).

During the COVID-19 crisis, viewer ratings for Channel 12 and Channel 13 plummeted. In October 2021, the N12 evening news edition had its lowest ratings for the past 20 years, with an average of 14.7 percent. On November 9, this figure for Channel 12 was even lower—only 12.8 percent. The Channel 13 evening news also had its weakest ratings in 15 years—only 7.8 percent, and on November 8, its ratings reached an all-time low of 5.9 percent (102). Of course these low ratings have many causes and interpretations. But as Gideon Dokov indicated in what was practically the only opinion piece written on this topic, “in response to the unreliable and professional coverage of the pandemic, the average viewer may have come to the conclusion that there is no reason to assume that coverage in other fields is any better. The insight that in at least one area the news channels continue to sell damaged goods and refuse to acknowledge this or to apologize led viewers to question the quality of goods on other shelves and ultimately to abandon the store. The tribal fires were extinguished by the viewers” (102).

### Study Limitations

One limitation of this study is that it only examined coverage of the vaccination campaign issue during specific months and did not examine other topics and issues relevant to the COVID-19 crisis. In addition, this study did not attempt to and does not necessarily represent the public opinion in Israel regarding the variables examined. Another limitation is that the study only examined the newscasts on Channels 12 and 13 and not on other television channels or in the press, on the radio or *via* digital channels. Future research should include these additional sources. Moreover, it should entail in-depth interviews with journalists and interested parties (e.g., government ministry employees, industrial advisors and more) in order to obtain a more complete picture.

## Conclusion

During pandemics and disasters, the job of the media is to provide fully transparent, reliable and up-to-date information based on risk communication to enable the public to make informed decisions during times of uncertainty (103).

The findings of this study lead to the following recommendations. First, the media should check, verify and validate all information originating from the Ministry of Health, and from any other source as well. The media should not report this information without conducting an independent journalistic investigation. Second, the media and the Ministry of Health should exercise caution in using the fear appeal strategy and the attribution of responsibility strategy, for these strategies may generate a boomerang effect among the public, leading to a lack of trust and a lack of cooperation.

As the watchdog of democracy, the news should function as a professional and objective source that criticizes government systems if necessary and strives to uncover the truth throughout the crisis. Public trust, which is so essential during such a crisis, can be achieved only if the news channels provide reports and meaningful journalistic investigations that challenge the system. By doing so, they can help fight conflicts of interest that divert management of the crisis from the professional health field to the political-economic arena.

## Data Availability Statement

The raw data supporting the conclusions of this article will be made available by the authors, without undue reservation.

## Author Contributions

AG-E conceptualized and supervised the study, set the criteria for analysis, performed the analysis of the data, reviewed the literature, and wrote the first draft of the manuscript. RH and RC collected the materials and participated in the analysis of the data. All authors reviewed and modified the article, contributed to the article, and approved the submitted version.

## Conflict of Interest

The authors declare that the research was conducted in the absence of any commercial or financial relationships that could be construed as a potential conflict of interest.

## Publisher's Note

All claims expressed in this article are solely those of the authors and do not necessarily represent those of their affiliated organizations, or those of the publisher, the editors and the reviewers. Any product that may be evaluated in this article, or claim that may be made by its manufacturer, is not guaranteed or endorsed by the publisher.

## Footnotes

^1^Note that in the Hebrew media discourse in Israel the word “corona” was the prevalent term used in the context of the COVID-19 pandemic.
